# Dissecting immune cell stat regulation network reveals biomarkers to predict ICB therapy responders in melanoma

**DOI:** 10.1186/s12967-021-02962-8

**Published:** 2021-07-08

**Authors:** Jingwen Wang, Feng Li, Yanjun Xu, Xuan Zheng, Chunlong Zhang, Congxue Hu, Yingqi Xu, Wanqi Mi, Xia Li, Yunpeng Zhang

**Affiliations:** College of Bioinformatics Science and Technology, Harbin Medical University, Harbin, 150081 China

**Keywords:** Immune single cells, Regulation networks, Immunotherapy

## Abstract

**Background:**

Immunotherapy is a revolutionary strategy in cancer therapy, but the resistance of which is one of the important challenges. Detecting the regulation of immune cells and biomarkers concerning immune checkpoint blockade (ICB) therapy is of great significance.

**Methods:**

Here, we firstly constructed regulation networks for 11 immune cell clusters by integrating biological pathway data and single cell sequencing data in metastatic melanoma with or without ICB therapy. We then dissected these regulation networks and identified differently expressed genes between responders and non-responders. Finally, we trained and validated a logistic regression model based on ligands and receptors in the regulation network to predict ICB therapy response.

**Results:**

We discovered the regulation of genes across eleven immune cell stats. Functional analysis indicated that these stat-specific networks consensually enriched in immune response corrected pathways and highlighted antigen processing and presentation as a core pathway in immune cell regulation. Furthermore, some famous ligands like SIRPA, ITGAM, CD247and receptors like CD14, IL2 and HLA-G were differently expressed between cells of responders and non-responders. A predictive model of gene sets containing ligands and receptors performed accuracy prediction with AUCs above 0.7 in a validation dataset suggesting that they may be server as biomarkers for predicting immunotherapy response.

**Conclusions:**

In summary, our study presented the gene–gene regulation landscape across 11 immune cell clusters and analysis of these networks revealed several important aspects and immunotherapy response biomarkers, which may provide novel insights into immune related mechanisms and immunotherapy response prediction.

**Supplementary Information:**

The online version contains supplementary material available at 10.1186/s12967-021-02962-8.

## Background

The majority of skin cancer-relevant deaths are accounted by melanoma [1, 2]. Only 23% of metastatic (stage IV) melanoma patients survived over 5 years after diagnosis [1]. Immunotherapy raised a revolutionary weapon against cancer [3–5]. Notably, anti-CTLA4 and anti-PD-1 inhibitors, ipilimumab and nivolumab, have achieved great increase in clinical benefit for carcinomas like metastatic melanoma [6–8]. Even though oncology is being revolved by the remarkable success of ICB therapies, the majority of patients received immune checkpoint blockade (ICB) therapies unfortunately do not benefit from the treatment [9–12]. Primary and acquired resistance also obstruct long-term curative-effect in patients with metastatic melanoma [9].

ICB therapy provided a great sense to investigate immune cell interactions of melanoma [13]. It is an urgent need to practice regulation analysis and data mining among immune cells under context of on- or post-therapy in melanomas. By profiling of single immune cells in baseline and on- or post-therapy samples in melanoma patients treated with checkpoint therapy, Hacohen et al. has defined 11 CD45+ immune cell stats which were associated with response to ICB therapy in metastatic melanoma [14]. These ICB therapy related CD45+ immune cell stats enable construction of regulation networks for different immune cell stats.

Currently, primary biomarkers of ICB therapies such as tumor mutational burden (TMB) and programmed death ligand 1 (PD-L1) expression [7, 15] have performed rough immunotherapy selection, and new biomarkers (e.g., eosinophilic count) show associations toward poor or longer survival [16]. For predicting of response to therapy, there are several studies attempt to inquire into alterations in expression of the PD-1/PD-L1 immune inhibitory axis or tumor microenvironment in patients with melanoma [8, 15]. Also, some studies focused on heterogeneities of individual cells by single-cell RNA sequencing [14, 17]. However, these researches have thus far provided only a limited understanding for immunotherapy response. Novel potential biomarkers and prediction models are still urgently needed.

Here, we constructed regulation networks based on specific immune cell stat, dissected transcriptome features of multiple single-cell cluster binding pathways and investigated predictive capability of single-cell-based network model. Our observation showed that ligands and receptors in immune cell related regulation networks have selective power of therapy response in patients receiving ICB inhibitors.

## Materials and methods

### Materials

*CD45+ single cell sequencing* Immune cell high throughput sequencing matrix of melanoma we used in this study was accessible in GEO database (GSE120575) which profiled 16,291 CD45+ immune cells from 48 tumor samples [14].

*Pathways* As reference pathways of our networks, BioPAX [18] level 3 integrated pathways were downloaded from https://www.pathwaycommons.org/archives/PC2/v10/. The common pathways contain 2,374,707 interactions of 32,875 participants integrated as 13 types (e.g., controls-expression-of, in-complex-with).

*mRNA sequencing of bulk tumor samples* mRNA expression of 56 MAGE-A3 checkpoint inhibitor treated melanomas was downloaded from GEO database (GSE35640). And twenty-two of these patients had complete or partial response to immunotherapy [19].

*Others* Homo species transcription factors (TF) were downloaded from AnimalTFDB [20] (http://bioinfo.life.hust.edu.cn/AnimalTFDB/#!/). Paired ligands and receptors were obtained from FANTOM [21] (http://fantom.gsc.riken.jp/5/suppl/Ramilowski_et_al_2015/) database.

### Methods

#### Integrating networks of melanoma immune cells

Each of the 16,291 immune cells was previously assigned into one of 11 unsupervised clusters according to the study of Sade-Feldman et al. [14]. Sade-Feldman M et al. applied the k-means algorithm to classify CD45+ immune cells. They used all genes with variance > 6 (about 4000 genes) to identify the unsupervised clustering of immune cells. They applied the k-means algorithm testing k = 3…15, and when k = 11, the clusters are most robust based on the 100 iterations in which they removed 10% of the cells randomly. Among those 11 clusters, C1 and C2 tend to be enriched by B cells, C3 and C4 are myeloid clusters and C6 and C9 are T cell clusters [14]. For each cluster, we constructed an immune cell cluster specific network considering three following aspects, in other words, two genes are considered to have an interaction relationship in our network and need to meet the following three conditions, a) each of the two genes must transcript in more than 1% immunity cells; b) there must be an interaction type in BioPAX pathways between them; c) co-expressed p-value (spearman correlation test) of these two genes must under 0.05 (Additional file 9: Figure S1).

**Fig. 1:**
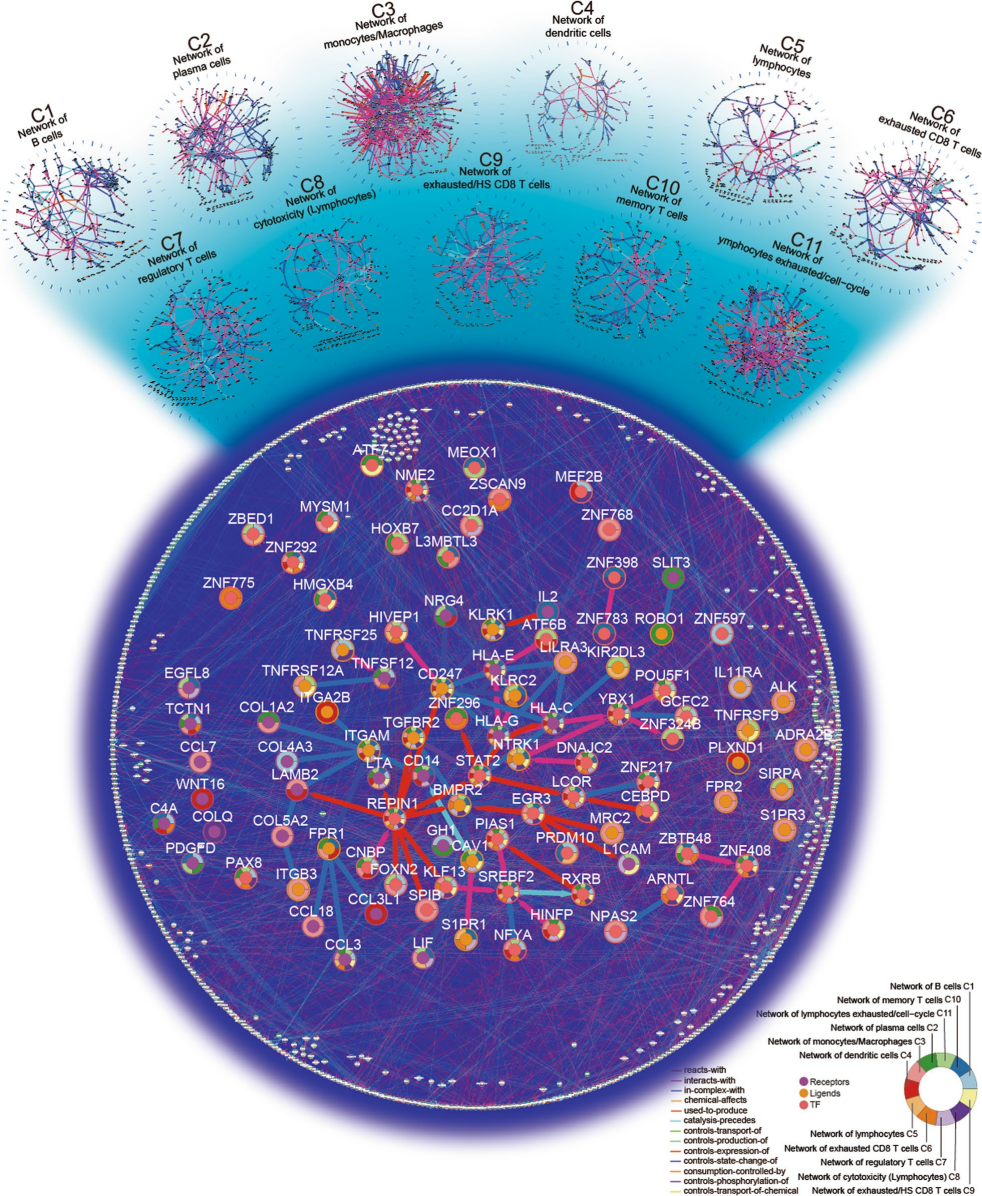
Global pathways of immunity single cell clusters under melanoma. Magnified nodes are TF, ligands and receptors; Bold edges are interaction among them. Float circle charts over nodes are colored by the 11 immuno-networks

We combined 11 immunity cell cluster specific networks (Additional file 8) into regulation networks and annotated them with TFs, ligands and receptors (Fig. 1, Additional file 9: Figure S1).

#### Topological properties evaluation of cell cluster specific networks and regulation networks

According to Barabási et al. [22, 23] and Zhang et al. [24], real biological gene interaction lies in scale-free networks, and degree distributions of genes in the network should comply with power law distribution (Eq. 1). We fit liner model for logarithm transformed gene degrees(X) and their distributions(Y) in a derived formula (Eq. 2) where a and b are coefficients to fit.

1 $$\mathrm{Y}=\mathrm{a}{X}^{-b}$$

2 $${log}_{10}Y=-b{log}_{10}X+{log}_{10}a$$

#### Identifying differently expressed genes

We divided cells into four groups: (1) cells from pre-treatment responders; (2) cells from pre-treatment non-responders; (3) cells from on-treatment responders; (4) cells from on-treatment non-responders. Then we calculated two measurements for responder and non-responder groups to access different expression—fold changes and Wilcox test p values. Finally, we used twofold and 0.05 significance as thresholds to define differently expressed genes.

#### Immunotherapy decision classification based on regulation networks

We investigated the predictive power of regulation networks in immunotherapy resistance by following steps:

1. fit logistic models—ligands and receptors in regulation networks were used as origin variables for model fitting in train data sets (half of GSE120575 samples selected randomly). We used “glm” function in R package “stats” to fit logistic models;
2. choose variables—formula-based model auto-selection were applied to select a subset of ligands and receptors along with their coefficients that can optimally guide therapy outcomes. We used “step” function to auto-select the logistic models and “predict” function to access prediction power of selected model;
3. test precision—variable sets we chosen in step 3 were tested in another half of GSE120575 samples. The “predict” function was used to calculate AUC in test data sets;
4. repeat circulation—step 1 to 3 were repeated 10,000 times.

Finally, the AUCs in above steps were used as standards to access prediction power of ligands and receptors in regulation networks.

## Results

### Regulation networks of immunity cell stats in melanoma

Tumor cell-intrinsic heterogeneity shapes the immune cell infiltration and influences the outcome of immunotherapy [25], and various metabolic pathways orchestrate the behavior of tumor-infiltrating immune cells, which are related to enhancing of antitumor immunity and immunotherapy [26]. Moreover, distinct CD45+ cell clusters revealed by single-cell RNA-seq were associated with clinical outcome of ICB therapies and reflected by different identifiers [14]. Thus, co-expression of pathway genes in multiple cell stats could offer cellular immunity regulation. Herein, we constructed immunity networks based on BioPAX pathways for previously defined CD45+ cell clusters [14] and combined them into regulation networks (Fig. 1, Additional file 9: Figure S2, Method Details). We fitted power law models for degree distributions of cell cluster specific networks and regulation networks (Additional file 9: Figure S3, Method Details), and the R squares greater than 0.88 all over these models. Immune networks and regulation networks constructed by our pipeline are scale free and similar to the real biological networks.

In sum, seven hundred and ninety genes, including 26 receptors, 26 ligands, and 47 TFs, and 3048 interactions were recognized by the regulation networks. Notably, IL-2 receptor is a T cell stimulating cytokine [27], and it not only drives the expansion of T cells and the contraction phase of immune response [28], but also has an effect on cancer stem cells [29, 30]. Most importantly, low dose IL2 combined with other immunotherapy demonstrated benefit in patients with metastatic melanoma [31]. In C10, a memory T cell cluster, IL2 controls expression of KLRK1 which controls state change of ITGAM through CD247 (in complex with HLA-C, HLA-E and HLA-G in all 11 cell clusters). IL2 controlling ITGAM, which in complex with CD14 (IL2, ITGAM, CD14 are cell surface markers) [32], indicted that our framework efficiently highlighted regulation flows of immunity system in distinct and common immune cell stats. Construction of immune cell stat specific pathways could offer contributions for immunology and ontology, including immune therapy response related researches.

### Divergence regulation of overlap genes was revealed by network comparative analysis among multiple networks

**Fig. 2:**
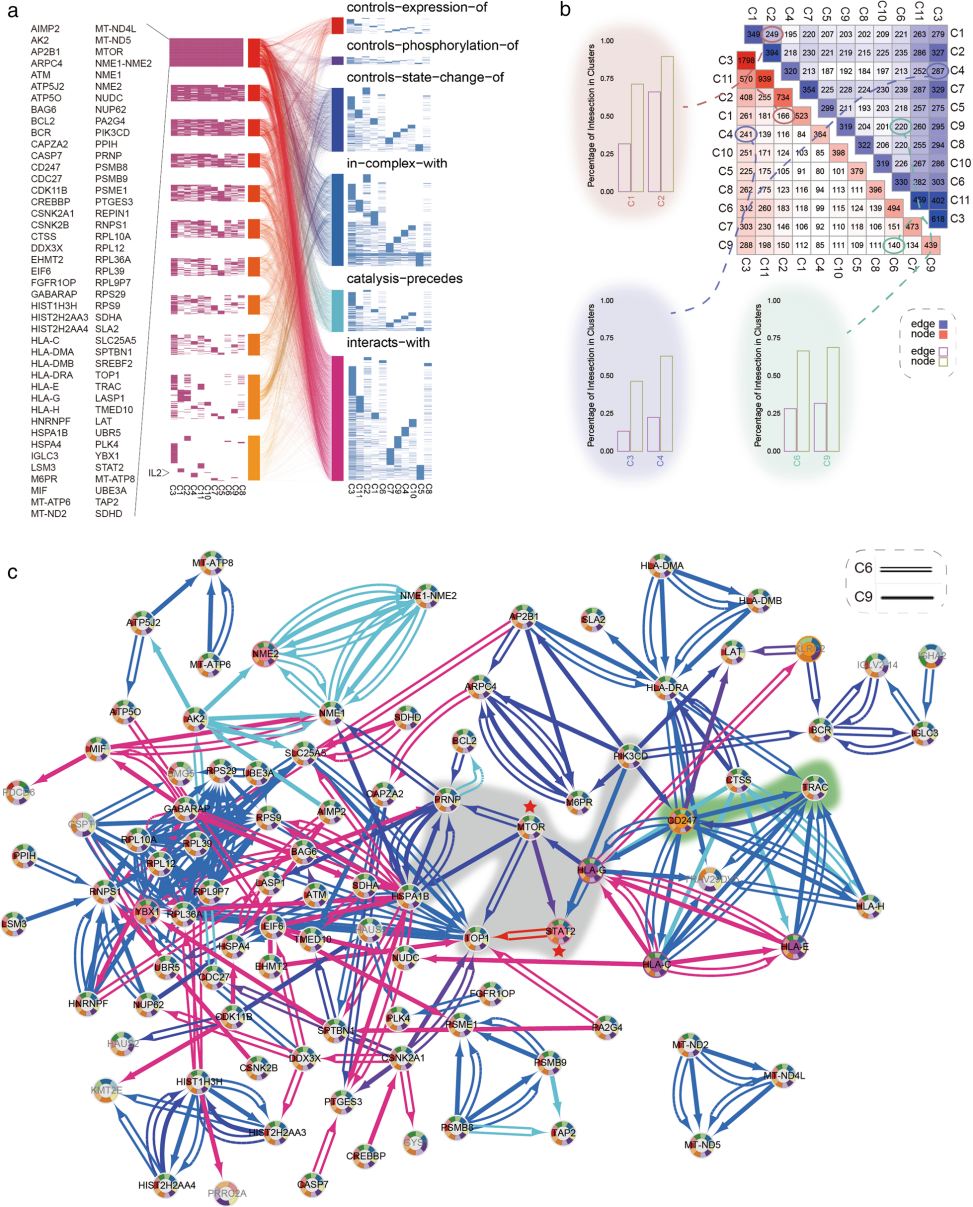
Cross-talk among 11 networks. **a** Heatmap showed genes(left) and interactions(right) that were contained by 11 immuno-networks and gaped by degree and interaction types. The middle of two plots showed association between genes and interactions among networks. **b**, **c** Overlap of edges and nodes among 11 networks. Intersection nodes and edges among networks and percentage of intersection in each network (**b**); Intersection regulation of networks constructed by cell cluster C6 and C9 (**c**)

Since our study highlighted different interaction flows of cluster specific networks, we accessed expression of genes and interaction pairs in distinct immune cell stats. There are several genes (86) such as major histocompatibility complex (HLA-C, HLA-DMA, HLA-DMB, HLA-DRA, HLA-E, HLA-G and HLA-H), CD247, cyclin dependent kinase (CDK11B), casein kinase 2 (CSNK2A1 and CSNK2B), FGFR1 oncogene partner (FGFR1OP) etc. that were activated in all 11 predefined T cell stats (Fig. 2a). However, consensus interactions among those common genes were partly observed. On the contrary, obvious inconsistent interaction pattern among common genes were discovered in B cell clusters (C1, C2), myeloid clusters (C3, C4) and CD8 T cell clusters (C6, C9) ([14], Fig. 2b, Additional file 9: Figure S4). Even though identifiers of distinct immune cell stat differ from the others, there are common genes, which exercise functions through different flows, that were activated in multi-networks. For instance, mechanistic target of rapamycin kinase (MTOR) state change was controlled by HLA-G in C9 but not in C6, and MTOR controls state change of HSPA1B in C9, TOP1 and PRNP in C6 (Fig. 2c). STAT2 interacts with different genes in C6 (HLA-G) and C9 (MTOR, PIK3CD). Moreover, a specific duplex interaction was observed in C9 (TRAC controls state change of CD247, CD247 in-complex-with TRAC, Fig. 2c). These results provide further evidence for our explanation that common genes from different cell stats can activate in variety ways in these cells.

Interleukin-2 (IL-2) antigen stimulates memory CD8(+) T cells production, and high relative IL-2 production in T cells of melanoma tend to perform memory CD8(+) T cells phenotype and superior proliferative capacity compared to cells with low IL-2 production [33]. In our research, IL2 and LIME1 specifically activated in a memory T cell cluster (C10). We also calculated different expression of IL2 in C10. The expression levels of IL2 gene is significantly expressed higher in cells from responders than cells from non-responders of on-treatment patients. Furthermore, several genes activated distinctly in different networks. For instance, PVRIG specialized in lymphocytes (C5), AGER, BHLHB9, CDK3, GNG8, IL11RA, PKIA, USP50 in regulatory T cells (C7). ARHGAP19, FNIP1 in exhausted/HS CD8 T cells (C9) and DECR2, SESTD1, ZNF775 in exhausted CD8 T cells (C6) etc. Our framework discovered different performance of immune related genes in cell cluster specific networks, which may lead to a functional nuance.

### Consensus functions across immune cell regulation networks

**Fig. 3:**
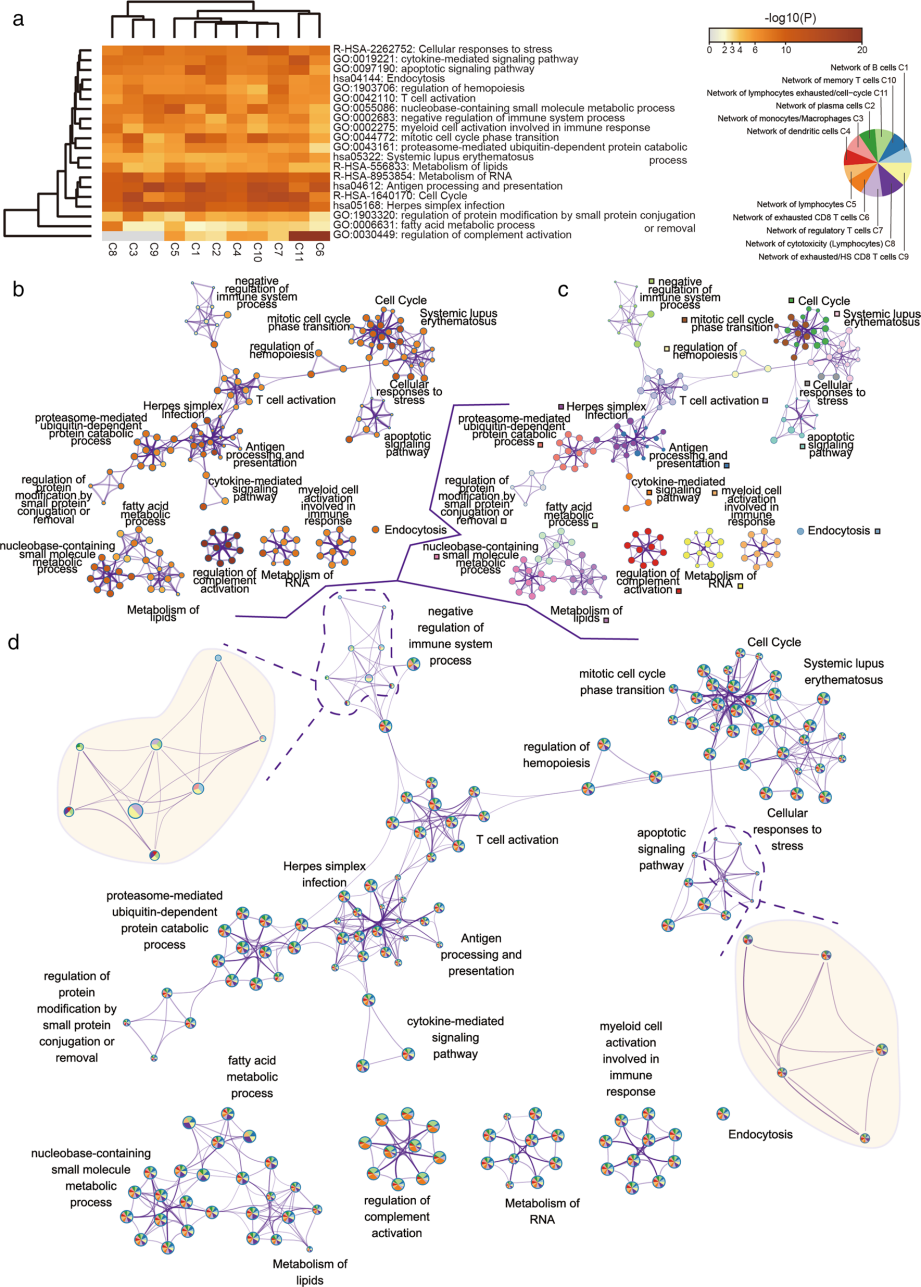
Gene sets functional enrichment of 11 networks. **a** Function enrichment of 11 networks and enriched p-values. **b**–**d** Enriched GO-terms colored by p-value (**b**), cluster (**c**) and counts (**d**). Gene sets enrichment analysis are performed by metascape online (http://metascape.org/)

To investigate functional relevance of cell cluster specific networks, we employed online pipeline metascape (http://metascape.org/gp/index.html) for enrichment analysis. We found that gene sets from plural networks enriched in consensus functions, for example, metabolism of RNA, antigen processing and presentation, cell cycle and herpes simplex infection, regardless of the different activation flows they presented (Fig. 3). Significantly, all networks partly enriched in several sub-terms of vital functions like T cell activation, cell cycle, cellular responses to stress, apoptotic signaling pathway, cytokine-mediate signaling pathway, herpes simplex infection, metabolism of RNA [34–38]. As for some functions, such as negative regulation of immune system process, were enriched by all networks but in different sub-terms. In addition, exhausted CD8 T cell cluster (C6) and lymphocytes exhausted/cell-cycle (C11) cluster significantly enriched in regulation of complement activation overall situation, yet regulation of complement activation was not enriched by monocytes/macrophage cluster(C3), cytotoxicity (lymphocytes) cluster (C8) and exhausted/HS CD8 T cell cluster (C9). Several sub-terms of negative regulation of immune system process were enriched by different networks despite all eleven networks were enriched in negative regulation of immune system process by a significant level. Computation of enriched function in immune cells could perform a systemic insight to understand mechanism of immune cell interaction.

### A core pathway: antigen processing and presentation

**Fig. 4:**
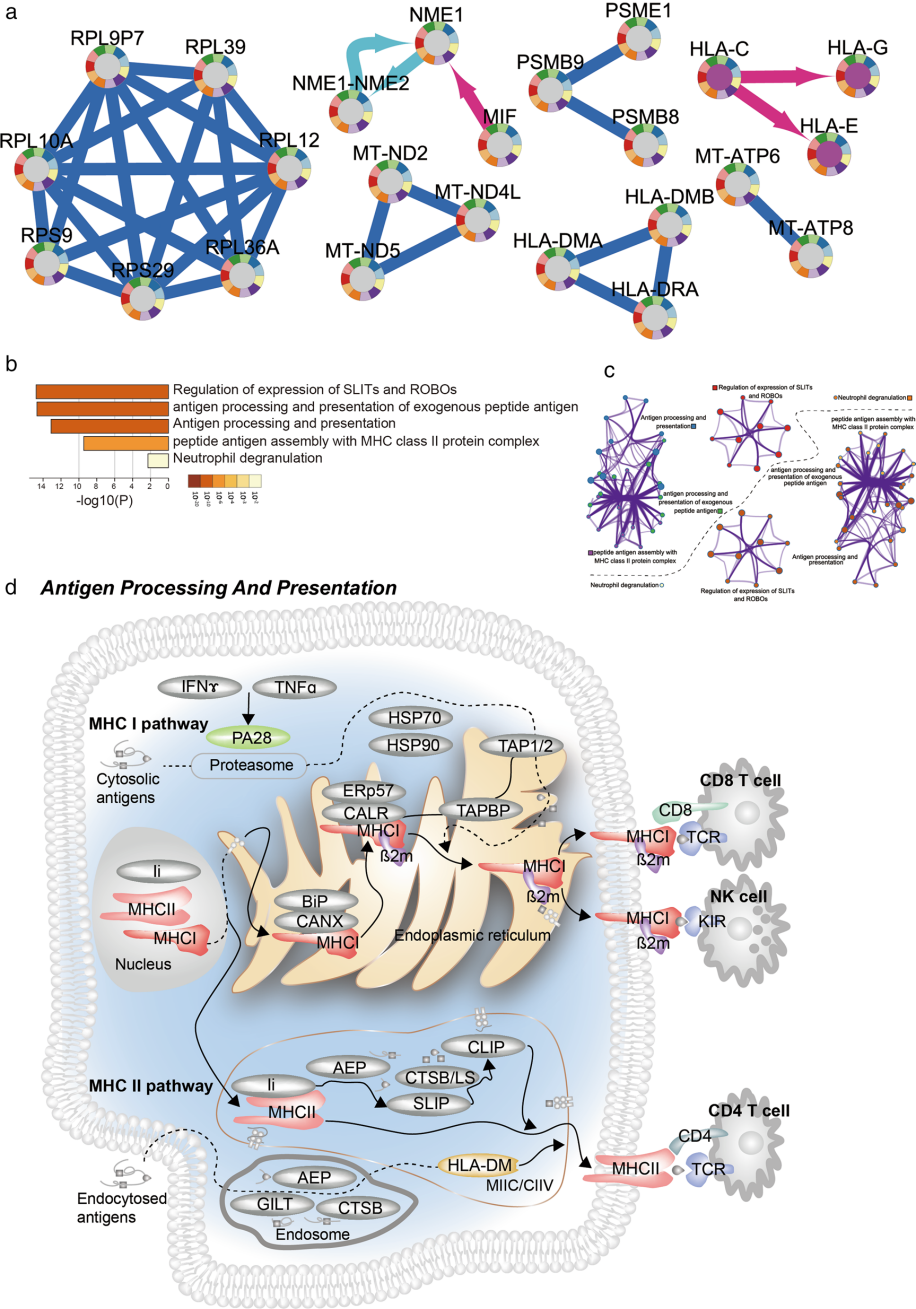
Shared sub-networks of 11 networks. **a** Shared interactions of 11 networks. **b** and **c** Function enrichment of shared genes. Gene sets enrichment analysis are performed by metascape online (http://metascape.org/). **d** A core pathway: Antigen Processing and Presentation. Colored nodes are enriched proteins

We discovered that shared topology of 11 networks covered two major histocompatibility complex unions (HLA-C, HLA-E, HLA-G; HLA-DMA, HLA-DRA, HLA-DMB), one ribosomal protein union (RPL10A, RPL9P7, RPL39, RPL12, RPL36A, RPS29, RPS9), one mitochondrially encoded NADH: ubiquinone oxidoreductase core subunit (MT-ND2, MT-ND4L, MT-ND5), one NME/NM23 nucleoside diphosphate kinase union (NME1, NME1-NME2) and one proteasome and proteasome activator subunit (PSME1, PSMB8, PSMB9) (Fig. 4a). Enrichment analysis of these shared topologic structures showed concurrent functions like regulation of expression of SLITs and ROBOs, antigen processing and presentation of exogenous peptide antigen, antigen processing and presentation peptide antigen assembly with MHC class II protein complex and neutrophil deregulation (Fig. 4b, c). Especially, subunits of the shared structures lie in two flows of antigen processing and presentation, MHC I and II pathways ([39], Fig. 4d), which play upstream roles of CD8 T cell killing target cells, regulation of NK cell activity (MHC I) and CD4 T cell cytokine production and activation of other immune cells (MHC II). According to Gene Ontology [40] enrichment analysis executed by metascape, three antigen related functions are interconnected among them, and the most significant enrichment is antigen processing and presentation.

### Key genes from networks were related with immunotherapy response at single cell level

**Fig. 5:**
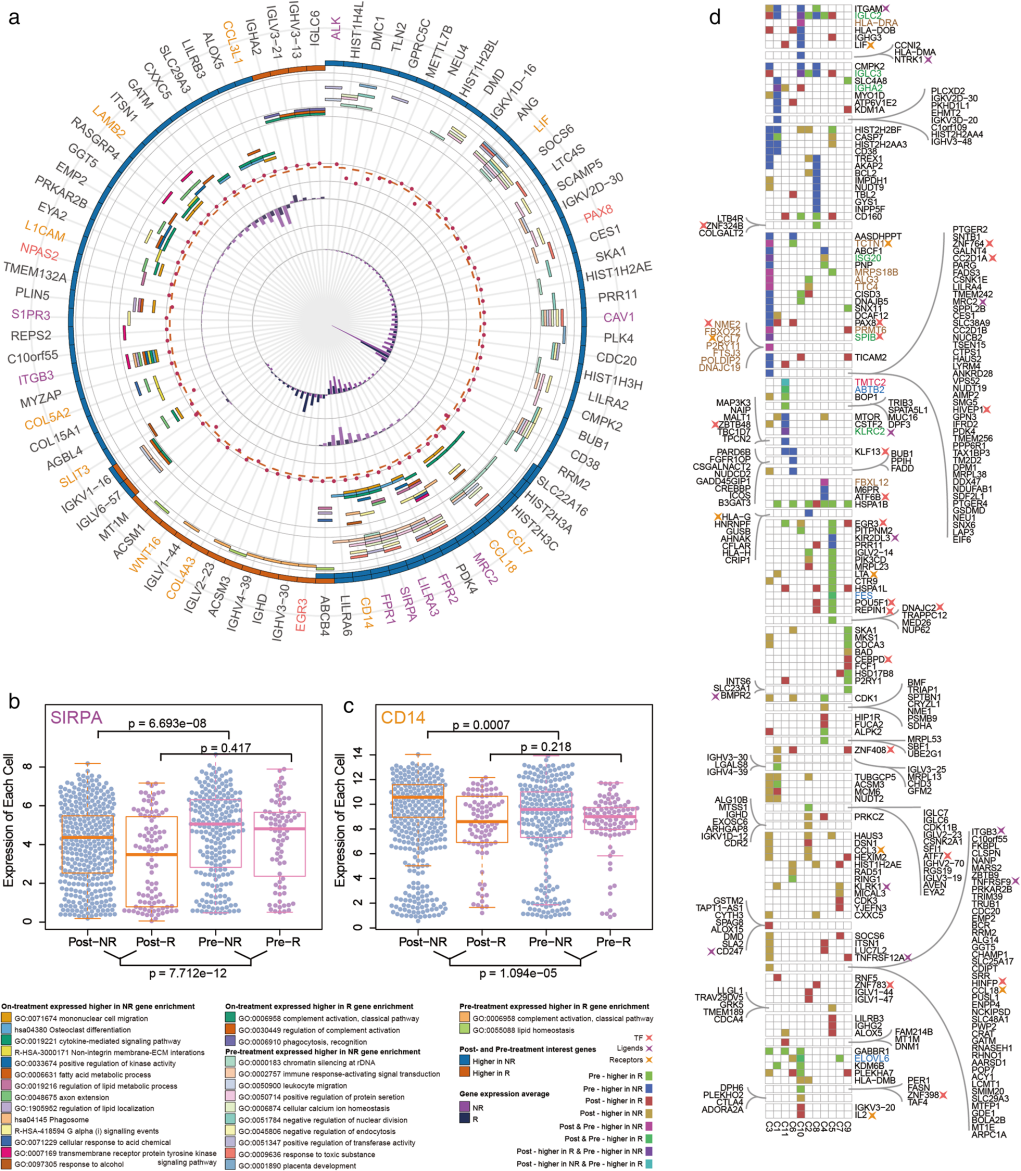
DEGs in global and local networks. **a** From the inside out: expression average (pre-treatment NR higher 3–0, pre-treatment R higher 0–1, on-treatment NR higher 1.5–0, and on-treatment R higher 0–1) and p-values (0.05–0) of DEGs. Gene sets function enrichment. on-treatment and pre-treatment DEGs. **b** and **c** SIRPA and CD14 expression in melanoma immunity single cells. **d** DEGs in 11 networks within cell clusters

Prior knowledge showed that a large number of genes appeared relevance with not only cancer occurrence but also treatment response, and these appearances came up in the single cell level as well [14, 19, 41–43]. Expectably, there were a batch of genes in our regulation networks which were differently expressed between responders and non-responders in both periods of treatment (pre- and on-treatment) regardless of immune cell stats (Fig. 5a). Functional analysis showed that genes expressed higher in non-responders from on-treatment samples were enriched in cytokine-mediated singling pathway, transmembrane receptor protein tyrosine kinase signaling pathway and so on, yet differently expressed genes from pre-treatment samples were enriched in immune response-activating signal transduction, response to toxic substance etc. Interestingly, several ligands and receptors differently expressed between responders and non-responders in pre- or (and) on-treatment samples, especially for a higher-in-non-responder set (CCL7, CCL18, MRC2, FPR2, LILRA3, SIRPA and CD14). Moreover, we detected a ligand, SIRPA, significantly expressed higher in non-responders and in pre-treatment samples. Consequently, SIRPA may play a resistance role in immunotherapy [44–46], and CAV1, SIRPA, CD14 were expressed higher in non-responders, may potentially support drug antagonism ([44–49], Fig. 5b, c).

Although we detected dysregulated genes in the single cell level, it still needs further inspection to uncover transcriptome changes in immune cell stats. Therefore, we next assessed differently expressed genes from cluster specific networks in corresponding CD45+ immune cell stats (Fig. 5d, Additional file 9: Figure S5-6). Our research suggested that ligand integrin subunit alpha M (ITGAM) was significantly upregulated in C3 from non-responders of on-treatment patients, in C1 and C10 from non-responders of pre-treatment patients, as well as in C8 from responders of pre-treatment patients. Regulating cell fate, ITGAM was dysregulated in multiple cell types (T cells, B cells et.), which may contribute to tumor cell survival in immunotherapy [50]. Ligand NTRK1, which expressed higher in pre-treatment non-responders, was dysregulated in C10, and IL2, a type I cytokine which can be associated with durable regression in metastatic melanoma and renal cell carcinoma [51], also was dysregulated in and only in C10 but expressed higher in on-treatment responders. Two major histocompatibility complexes, HLA-G and HLA-H, expressed higher in C2 from pre-treatment non-responders. Another major histocompatibility complex, HLA-DMB, expressed higher in both C2 and C10 in non-responders from on-treatment samples. Other famous genes, such as tumor necrosis factor receptor superfamily member 9 (TNFRSF9) and CD247 ligands, were also upregulated in C3 from (non-)responders of on-treatment patients, and another tumor necrosis factor receptor superfamily member (TNFRSF12A) was dysregulated in both C3 and C9. Thus, we have reason to suppose that some ligands and receptors may propose a new insight to understand drug sensitivity and resistance, as well as immunotherapy response.

### Ligands and receptors of regulation networks showed robust selective power in immunotherapy response

**Fig. 6:**
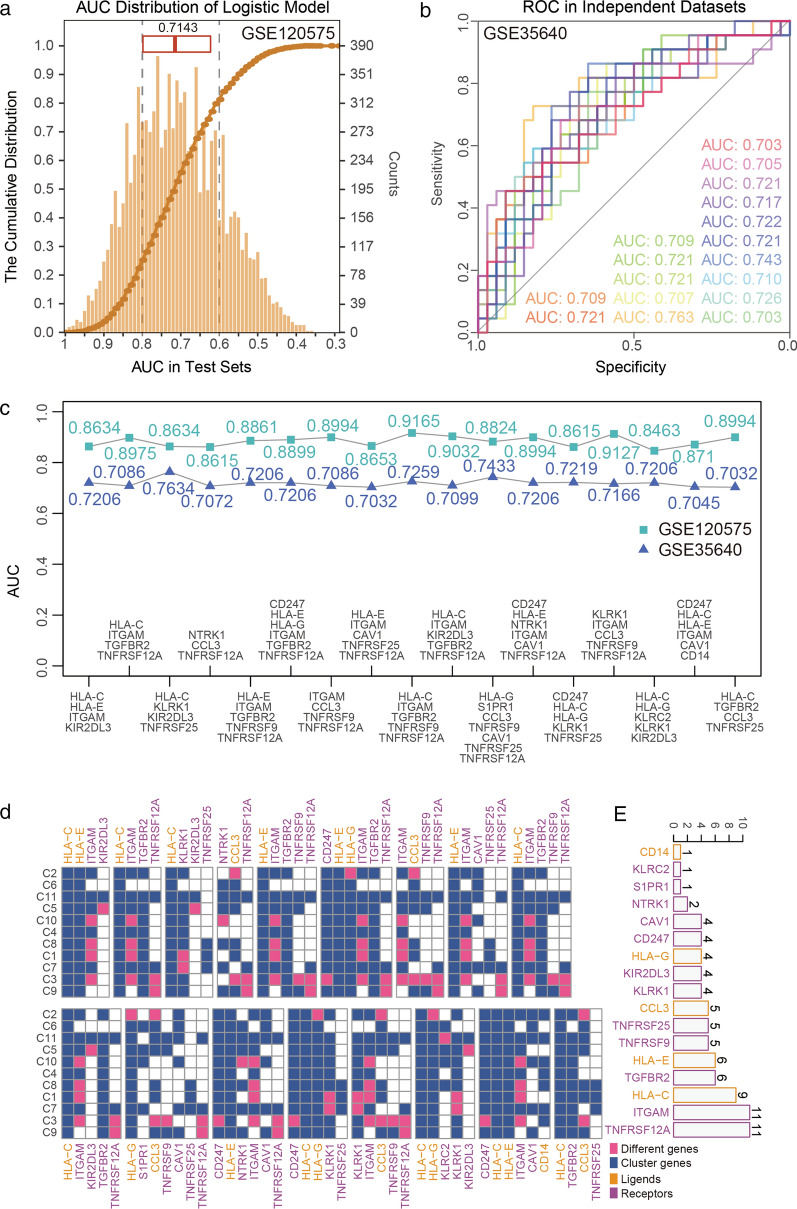
Immunotherapy resistance accuracy of immuno-networks. **a** AUCs in test data sets during model construction are showed in form of 1) Boxplot—0.25, 0.5, and 0.75 quantile; 2) cumulative distribution; and 3) Barplot—counts of AUC values. **b** ROCs of 17 promising immunotherapy predictors under independent validation. **c** AUCs of test data sets during model construction and independent data sets of 17 promising immunotherapy predictors. **d** 17 predictors activate in 11 networks. **e** Gene counts of 17 predictors

Some receptors can mediate functions of immune cells through distinct signaling pathways [52]. Changing of these receptors and corresponding ligands may lead unexpected immunotherapy outcomes. Our research also observed massive changes of gene expression of these proteins. Hence, we applied logistic regression model to select contribution features from ligands and receptors of regulation networks and access immunotherapy precision of featured gene sets. In the test data sets, medium AUC of 10,000 random is 0.7143, and most of the test AUCs are among 0.65 and 0.8 (Fig. 6a). Eventually, we identified 17 gene sets that associated with immunotherapy response. In an independent validation, GSE35640, all 17 gene sets performed AUC greater than 0.7 (Fig. 6b), which suggests robust selective power of ligands and receptors of regulation networks for immunotherapy response. This completed key gene analysis by providing gene sets and their scores which were able to construct classification of therapy response.

Several TNF receptor superfamily members were integral to the immune-response regulation by enhancing T-cell growth and dendritic-cell function. These proteins related to modulation of cellular functions, proliferation, survival or deaths [53]. Moreover, TNF receptors controlling TNF receptor signaling, which plays its role in inflammation and cell death, could determine the cellular fate [54]. Especially, TNFRSF12A (also known as TWEAK receptor, Fn14, or CD266) correlated with integrin β3 expression, which drives Glut3 expression, is associated with clinical outcome and tend to be responsible for inducing cachexia in tumors [55, 56]. We identified one TNF receptor leaded gene set, consisting of TNF receptor superfamily member 9/25/12A (TNFRSF9, TNFRSF25 and TNFRSF12A), sphingosine-1-phosphate receptor 1 (S1PR1), C–C motif chemokine ligand 3 (CCL3), caveolin 1 (CAV1) and major histocompatibility complex, class I, G (HLA-G), that predicted immunotherapy response with AUC up to 0.7433 in independent validation and 0.8824 in GSE12575 (Fig. 6c). Besides, CAV1 catalysis preceding of resistance related gene CD14 [49, 57], as well as supported a firm set predicting immunotherapy response (0.7045 in GSE35640, 0.871 in GSE120575) with CD247, HLA-C, HLA-E, ITGAM and CD14. Thus, TNFRSF12A, which works for 11 of 17 selected gene sets, cooperating with CAV1 and other ligands and receptors are key factors in immunity regulation and flexible immunotherapy response (Fig. 6d).

Additionally, other gene sets with major histocompatibility complexes (HLA-C, HLA-E, HLA-G), TNF receptors, ITGAM, CD247 and CD14 performed acceptable precision with AUC around 0.72 in the independent datasets and above 0.85 in GSE12575 as well. Furthermore, ITGAM, which regulates cell fate [50], also works 11 of 17 selected genes sets and HLA-C works in 9 genes sets (Fig. 6e). These genes not only activate in majority immune cells but also have a quality for prediction of immunotherapy response.

## Discussion

Great progress has been achieved in ICB therapy, yet therapy resistance must be considered in an actual treatment process. Consequently, it is important to investigate biomarkers of immunotherapy especially in single cell level. In this study, we constructed regulation networks across immune single cell types and established that differently expressed genes between cells from responders and non-responders. Some differently expressed genes coincided with ligands and receptors in immune cell specific pathways. Further analysis of ligands and receptors in regulation networks proposed prediction biomarkers for inhibitor response classifier in metastatic melanoma with ICB therapy. We trained logistics regression models to test prediction accuracy of biomarkers for immunotherapy response. Our results suggested 17 gene sets which could be useful for prediction of ICB therapy.

We found out that metabolism of RNA, antigen processing and presentation, cell cycle and herpes simplex infection networks were generally activated in all immune cell clusters but with different interaction flows. We also found that different sub-terms of negative regulation of immune system process were enriched by variety cell cluster specific networks even all the cell stats activated negative regulation of immune system process. Notably, we discovered a common topologic structure of all immune cell specific networks performing antigen processing and presentation function, which connected with antigen processing and presentation of exogenous peptide antigen and peptide antigen assembly with MHC class II protein complex. These three terms were essential for endogenous simulated immunity defense with cell surface MHC molecular carry and display viral peptides [58]. It requires an army of genes to coordinate for immune response and raise a weapon against tumor. MHC class I and class II molecules played a global relative role in presentation and processing of the antigen with its high polymorphic [59]. For MHC class I, we detected that HLA-C respectively interacted with HLA-G and HLA-E. And for MHC class II, we found a triple complex relationship among HLA-DMA, HLA-DMB and HLA-DRA. These results indicated that antigen processing and presentation may be a core functional region in immune cell regulation and proposed further explanation of immune response in ICB therapy.

We further revealed some key factors such as SIRPA, CD14, IL2, ITGAM and CD247 (differentially expressed between cells of responders and non-responders). These ligands and receptors tend to be associated with drug sensitivity and resistance, as well as immunotherapy response. Especially, IL2 controls expression of KLRK1 which controls state change of ITGAM through CD247. IL2 controlling ITGAM, which in complex with CD14 (both are cell surface markers, [32]), are specific in C10 and this type I cytokine can be associated with durable regression in metastatic melanoma and renal cell carcinoma [51]. In this study, biomarker gene sets detected by CD45+ immune single cells showed robust perspective power in ICB therapy response. Our results suggested that TNF receptors, MHC molecules, ITGAM, CD247 or CD14 leading gene sets can preciously distinguish the responders for non-responders in patients with melanoma that received ICB therapies. The accuracy of immune cell regulation network-based model may provide helpful guidance for precision medicine, as well as new understanding of immunotherapy response. Specifically, SIRPA and CD14 were upregulated in non-responders of both pre- and on-treatment patients. They both regulate TRIM27, and CD14 which contribute as a member of prediction gene sets in the logistics models. CD14, ITGAM, CD247 and MHC molecules not only significantly dysregulated between the responders and non-responders of patients with melanoma but also presented accuracy prediction of immunotherapy response. Several famous genes like MHC molecules (HLA-C/E/G), TNF receptor superfamily members (TNFRSF9/12A/25), ITGAM, CD14, CCL3 and CAV1 all performed dysregulation between responders and non-responders in the global or immune cell stat specific context, and they also provided great independent cooperation in prediction of immunotherapy response.

We identified 17 gene sets that associated with immunotherapy response. In an independent validation, GSE35640, all 17 gene sets performed AUC greater than 0.7. We compared 17 predictors identified by our analysis to ICB response biomarkers (PD-L1 and IFNG) which were widely used by clinical trials [60–63]. Our results showed that AUC of PD-L1 expression is 0.69 and AUC of IFNG expression is 0.75 (Additional file 9: Figure S7). Our results indicated that using ligands and receptors in the regulation networks to train decision models could provide a brand-new view for immunotherapy response, and these models could be potential guidance for precision medicine. We also tried to compare our predictor with TIDE [64]. However, the TIDE claimed no therapy responders for our independent data sets (Additional file 1: Table S1). In summary, our research could provide a new view for immune cell regulation mechanism and immunotherapy response prediction, and these models could be potential guidance for translational medicine and precision medicine. Moreover, we calculated different expression of biomarkers, which we claimed, between cell clusters (Additional file 9: Figure S8a). We found that these biomarkers different between single-cell types. We used CIBERSORT (https://cibersort.stanford.edu/) to perform deconvolution of cell types in bulk data GSE35640, and accessed immune score difference of CIRBERSORT cell type. We found that the cell difference of biomarkers in single cell level are connected with bulk data GSE35640, indicating a promising prospection of biomarkers we performed in precision medicine (Additional files 2, 3, 4, 5, 6, 7, 8).

## Conclusion

We constructed regulation networks across immune single cell types and established that differently expressed genes between cells from responders and non-responders. In sum, 790 genes and 3048 interactions, including 26 receptors, 26 ligands, and 47 TFs were recognized by the regulation networks. Some differently expressed genes coincided with ligands and receptors in immune cell specific pathways. Further analysis of ligands and receptors in regulation networks proposed prediction biomarkers for inhibitor response classifier in metastatic melanoma with ICB therapy. We trained logistics regression models to test prediction accuracy of biomarkers for immunotherapy response. In summary, our study provided single cell-based regulation networks in context of melanoma model with or without ICB therapy and revealed several gene sets consist of therapy response biomarkers which is efficient for construction of ICB therapy response classifier. These results could provide a new view for immunotherapy response prediction, and these models could be potential guidance for translational medicine and precision medicine.

## Supplementary Information

**Additional file 1.** Table S1.

**Additional file 2** Cluster names.

**Additional file 3** Expression of ligands and receptors.

**Additional file 4** Gencode basic.

**Additional file 5** Patient response.

**Additional file 6** Test codes.

**Additional file 7** Logistic codes.

**Additional file 8** Networks.

**Additional file 9** Supplementary figures.

## Data Availability

All data and codes in this study are available under proper request, please contact lixia@hrbmu.edu.cn. Our codes for AUC calculation on independent datasets are available in supplementary materials (Additional file 2, 3, 4, 5, 6, 7).

## Abbreviations

ICB: Immune checkpoint blockade
PD-L1: Programmed death ligand 1
TF: Transcription factor
IL-2: Interleukin-2
