# Supplementary material for: Dissecting immune cell stat regulation network reveals biomarkers to predict ICB therapy responders in melanoma
Source: J Transl Med. 2021 Jul 8;19:296. doi: 10.1186/s12967-021-02962-8 (PMC8265039; doi:10.1186/s12967-021-02962-8)
Supplement: Supplementary file 9 — Additional file 9 Supplementary figures. [file 12967_2021_2962_MOESM9_ESM.docx]

**Supplementary figures**


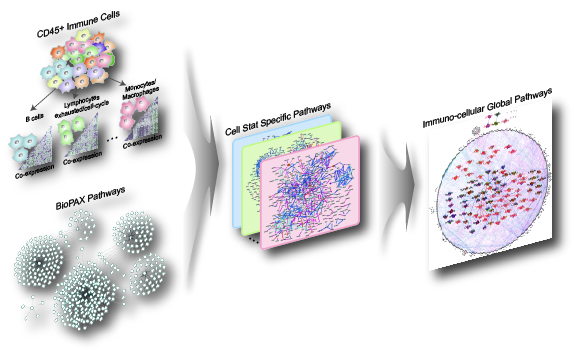


**Figure S1** Workflow describing construction of immuno-cellular global pathways


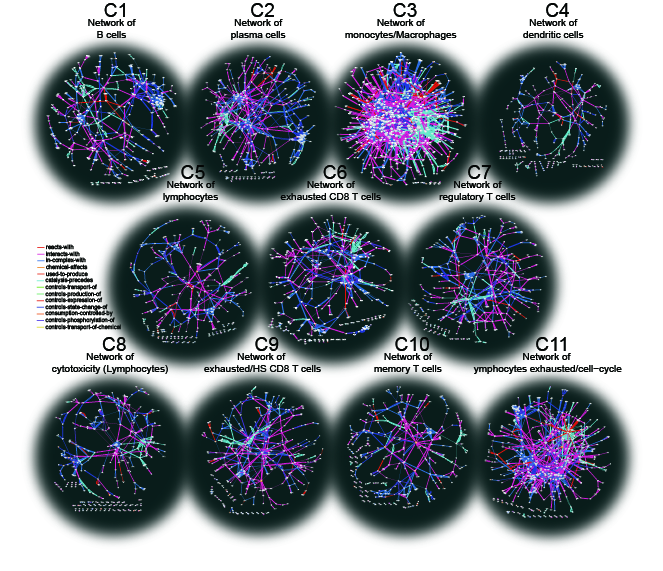


**Figure S2** eleven immuno-networks regarding immuno-cellular clusters


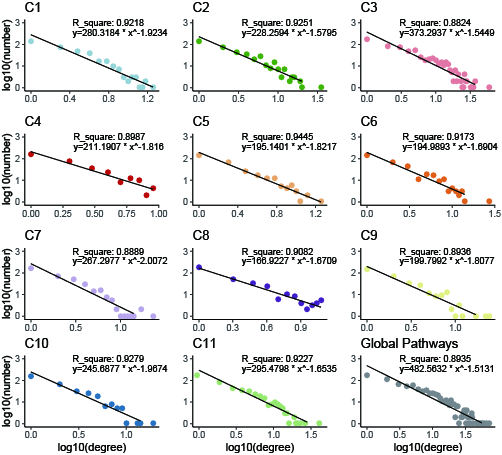


**Figure S3** Scale-free validation of all networks


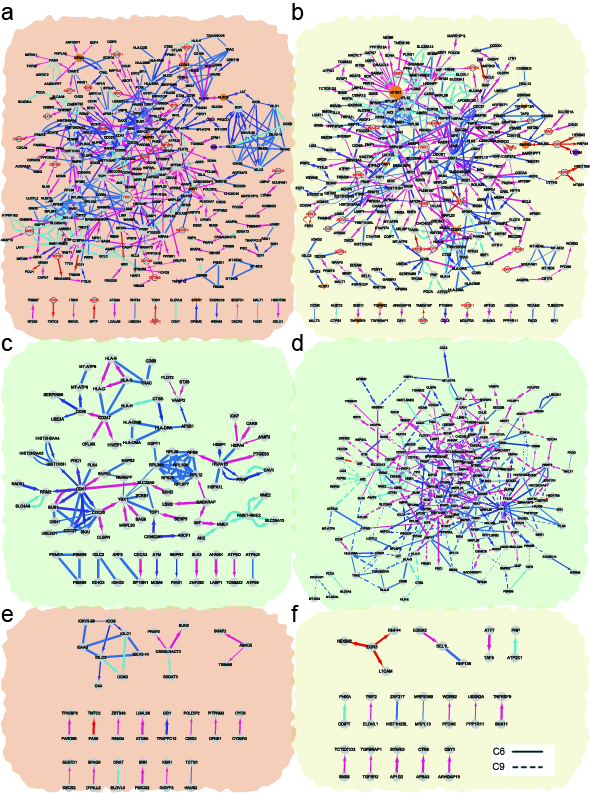


**Figure S4** Cross-talk of B cell networks (C1 and C2), T cell networks (C6 and C9), myeloid networks (C3 and C4)


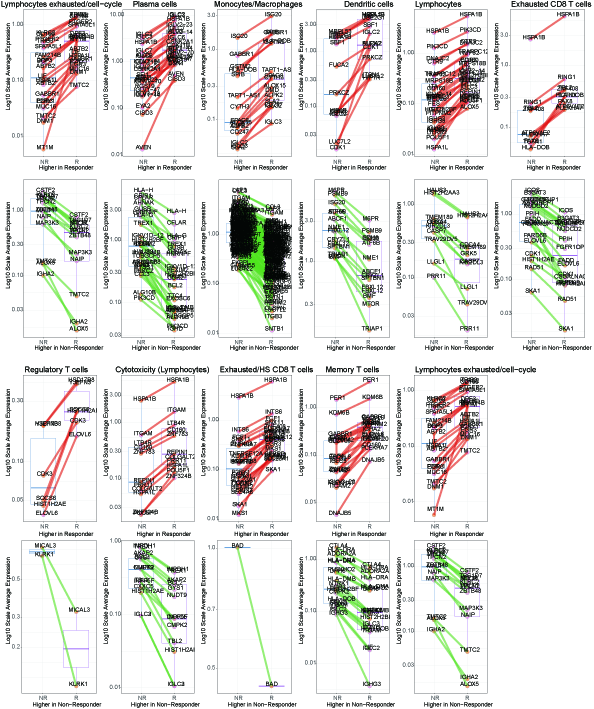


**Figure S5** Average expression of DEGs


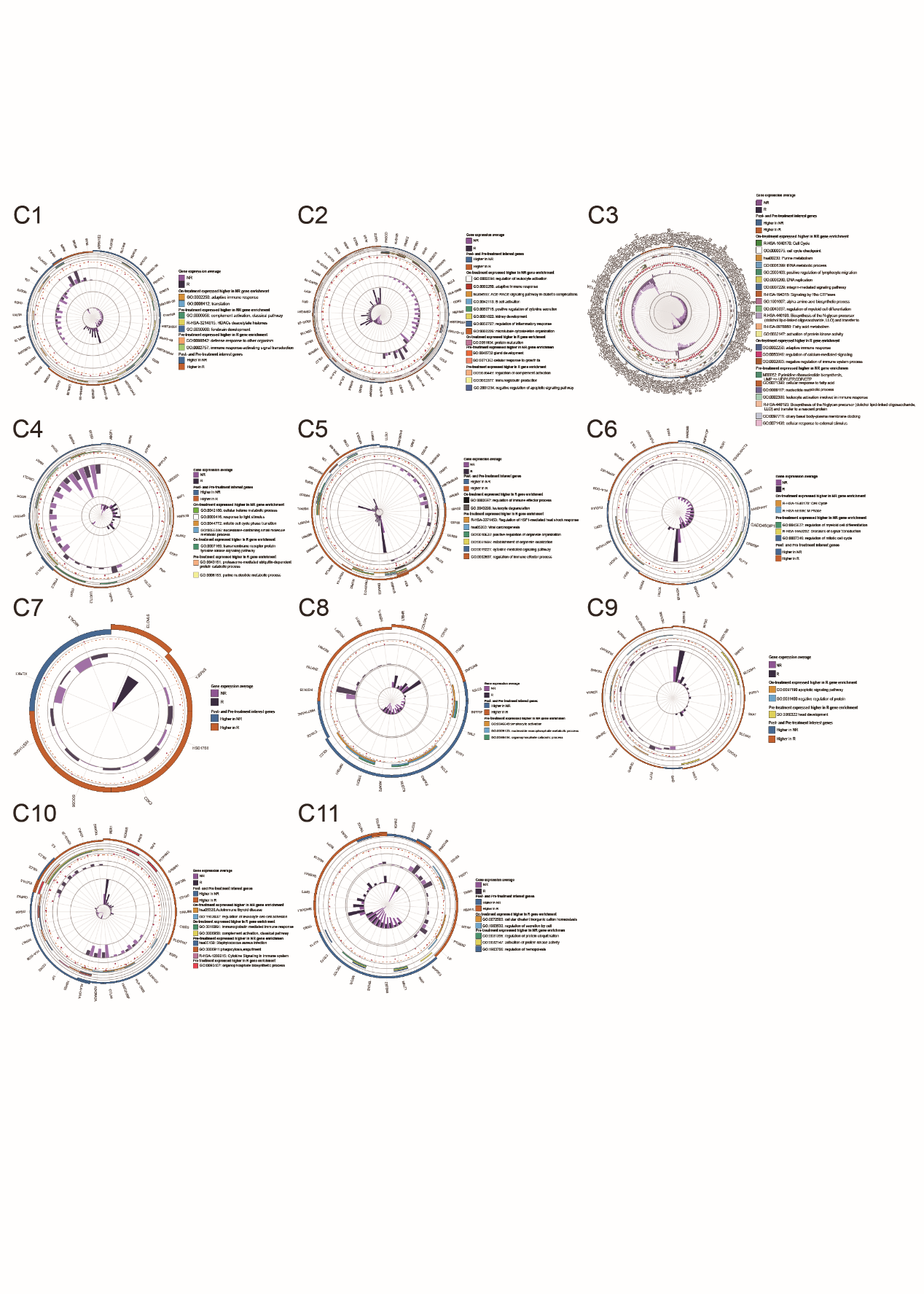


**Figure S6** Expression of DEGs in local networks


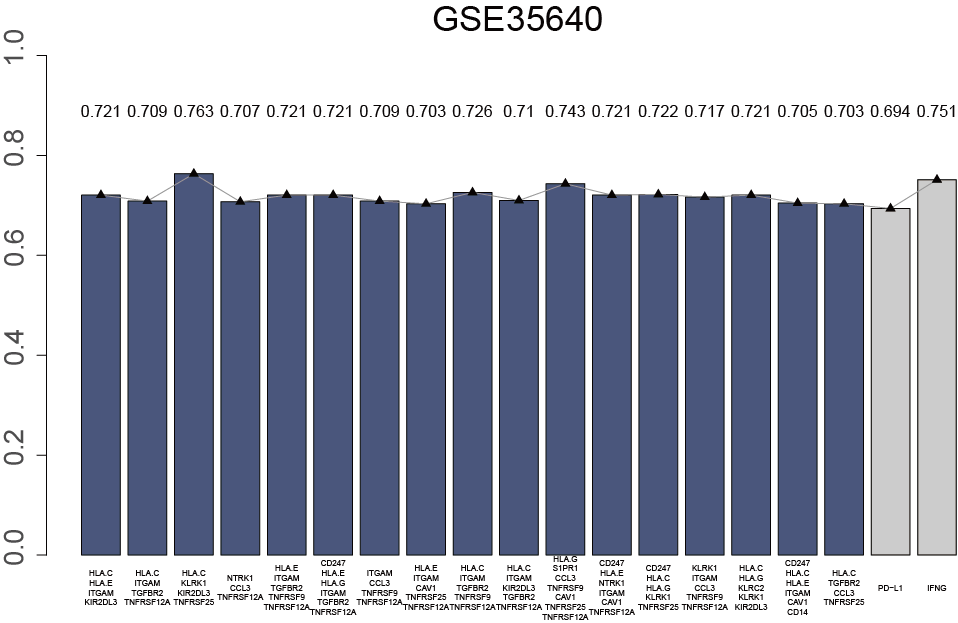


**Figure S7** Comparison of 17 predictors and immune checkpoint.


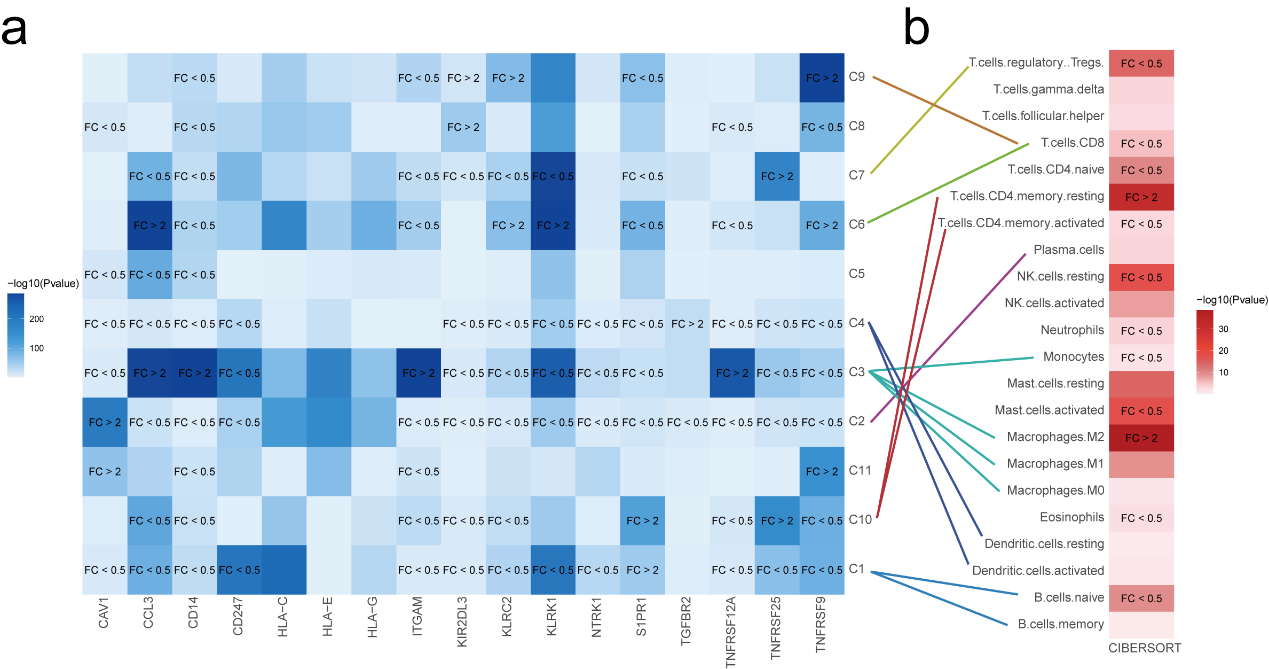


**Figure S8** Deconvolution of cell types in bulk data GSE35640. a) Expression difference between cell clusters of biomarkers. FC > 2 represent that the average of gene expression in one cell cluster is over two-fold higher than the other cell clusters; b) Immune score difference of CIRBERSORT in bulk data GSE35640. FC > 2 represent that the average of immune score in one cell type is over two-fold higher than the other types.
